# Treatment of juvenile bullous pemphigoid with lebrikizumab

**DOI:** 10.1111/ddg.15831

**Published:** 2025-07-02

**Authors:** Marcel Wittenberg, Farzan Solimani, Amrei Dilling, Rawan Snobar, Kamran Ghoreschi, Marisa Klemp, Kerstin Kusch

**Affiliations:** ^1^ Department of Dermatology Venereology and Allergology Charité – Universitätsmedizin Berlin Corporate Member of Freie Universität Berlin Humboldt‐Universität zu Berlin Berlin Institute of Health Berlin Germany

**Keywords:** Juvenile bullous Pemphigoid, Lebrikizumab, pediatric dermatology, interleukin 13, monoclonal antibodies

Dear Editors,

Juvenile bullous pemphigoid (BP) is a rare blistering disease that occurs in infants and school‐aged children. As with BP in adults, IgG autoantibodies directed against BP180 and/or BP230, two important hemidesmosomal components, trigger the disease.^1^ Clinically, juvenile BP resembles BP and is characterized by erythematous plaques, papules, and blisters accompanied by severe pruritus, but it also shows specific clinical patterns such as frequent acral involvement.^1^, ^2^ Juvenile BP is treated with topical and/or systemic steroids, although severe cases require steroid‐sparing agents such as dapsone, azathioprine, or methotrexate.^1^ The use of systemic immunosuppressants is associated with adverse effects and necessitates regular monitoring of hematologic parameters as well as liver and kidney function.^3^ Monoclonal antibodies (mAbs) targeting cytokines are generally better tolerated and require less frequent laboratory monitoring compared to oral immunosuppressants. MAbs that target type 2 cytokines are approved for treating atopic dermatitis (in both adults and children) and prurigo nodularis, but not for BP, where type 2 cytokines appear to be critical factors in disease pathology.^4^ The IL‐4 receptor alpha blocker dupilumab showed efficacy for BP in case series and is in phase III development.^5^ The anti‐IL‐13 mAb tralokinumab also showed encouraging results in a recently published case series.^6^ Here, we demonstrate that lebrikizumab, a humanized anti IL‐13 mAb effectively counteracts juvenile BP.

A 16‐year‐old boy was referred to our department due to the development of erythematous, pruritic plaques (VAS 9/10) on the trunk and arms and the presence of tense blisters on the feet (Figure 1a–d). The patient also had a positive history of Th2 diseases such as atopic dermatitis, allergic rhinitis, and asthma. Due to the clinical appearance with tense blisters we performed two skin biopsies for histology and direct immunofluorescence (DIF). Histology showed a mild acanthosis, spongiotic dermatitis with subepidermal blister formation and an eosinophil‐ and neutrophil‐rich infiltrate. DIF revealed strong linear IgG deposition along the basement membrane (Figure 1e,f). Laboratory tests showed mild eosinophilia (11.5%) and highly elevated total IgE levels (> 5,000 kU/l), indicating an atopic diathesis. In the patient's serum we could detect high titers of anti‐BP180 antibodies (> 200 U/ml) by Enzyme‐linked Immunosorbent Assay (ELISA). These results confirmed the diagnosis of a juvenile BP in a patient with pre‐existing atopic dermatitis. We initially started a topical treatment with betamethasone propionate twice daily and systemic doxycycline 100 mg daily combined with oral prednisone (initially 1 mg per Kg body weight). After four weeks of treatment, only slight clinical improvement was observed (VAS 8/10). Based on immunologic considerations about the immunopathogenesis of BP and the presence of concomitant Th2 diseases and the desire to avoid the use of steroid‐sparing agents at a young age, we decided to initiate a treatment with lebrikizumab (atopic dermatitis regimen) in combination with topical betamethasone propionate. This approach led to a rapid reduction in blistering and the disappearance of skin lesions and itching (VAS 0/10). After a few weeks, only post‐inflammatory hyperpigmented lesions were visible without signs of further ongoing inflammation (Figure 1g–i). The patient was followed up for 20 weeks without signs of relapse or worsening. Recent studies have shown that a history of atopic dermatitis and allergic rhinitis increases the risk of developing bullous pemphigoid; affected patients often require systemic treatment.^7^ Lebrikizumab is approved for the treatment of atopic dermatitis in adults and adolescents aged 12 years and older and demonstrates a more favorable safety profile compared to steroid‐sparing agents. This mAb has a high affinity and slow dissociation rate from IL‐13, a pivotal cytokine in BP immunopathogenesis.^4^ IL‐13 is the most prominently expressed T helper type 2 (Th2)‐associated cytokine in both the blood and skin of patients with bullous pemphigoid.^4^

**FIGURE 1:**
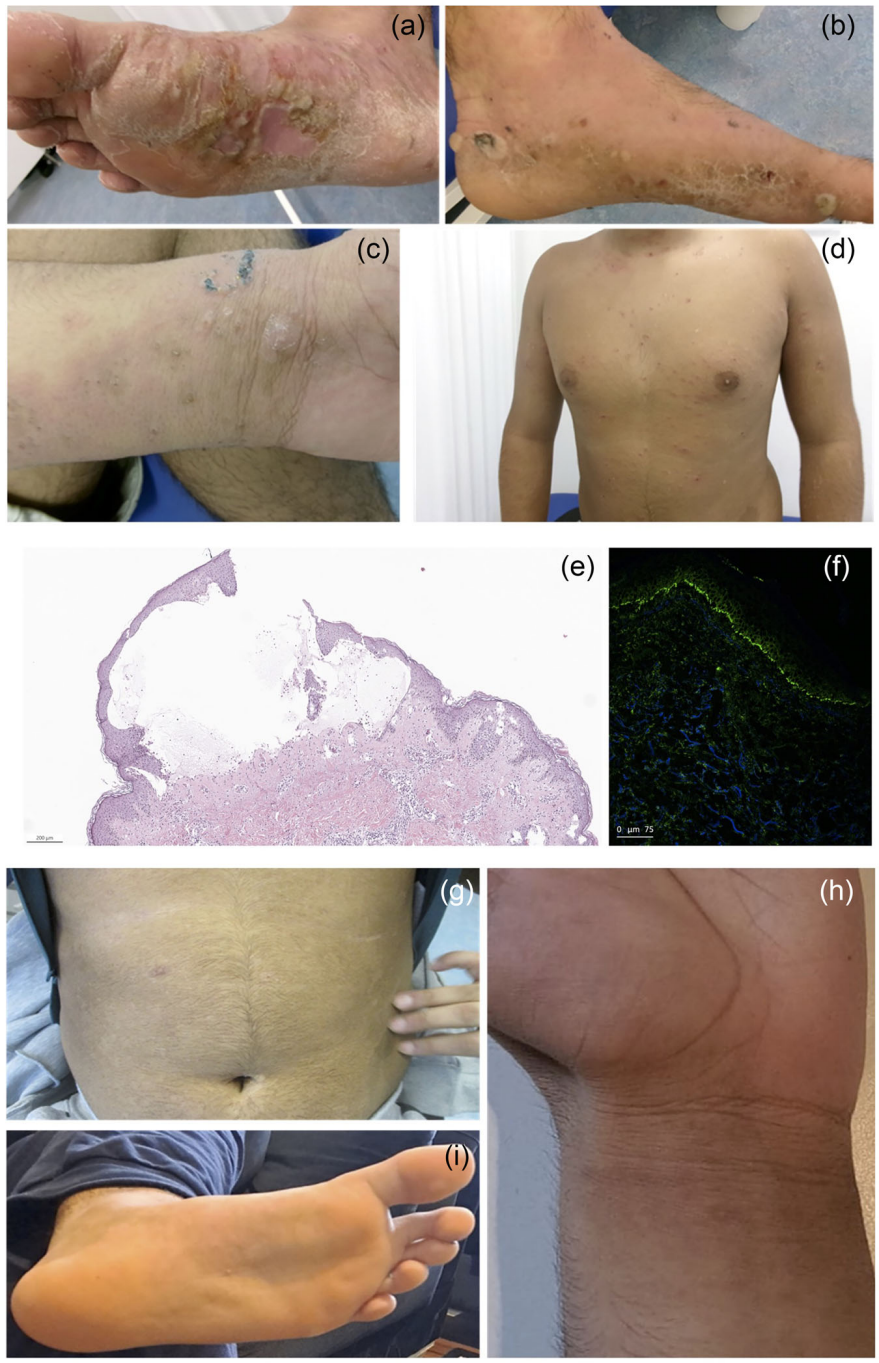
(a–d) Clinical presentation of a 16‐year‐old boy affected by juvenile bullous pemphigoid with disseminated erythematous papules and plaques, and tense blisters on the extremities. (e) Histological examination (hematoxylin and eosin staining) shows the presence of a subepidermal skin detachment and the presence of spongiosis in conjunction with a rich cellular infiltrate (hematoxylin‐eosin stain, scale bar: 200 µm). (f) Direct immunofluorescence with linear deposition of IgG along the junction zone. (g–i) Clinical effect of lebrikizumab after 20 weeks of administration. Lebrikizumab led to a complete resolution of skin lesions with minimal post‐inflammatory lesions.

This report underlines the central role of IL‐13 in the immunopathogenesis of BP and further supports the concept that anti IL‐13 approaches may contribute to disease control.

## CONFLICT OF INTEREST STATEMENT

None.
